# Acemannan Gels and Aerogels

**DOI:** 10.3390/polym11020330

**Published:** 2019-02-14

**Authors:** Daniel Alonso Miramon-Ortíz, Waldo Argüelles-Monal, Elizabeth Carvajal-Millan, Yolanda Leticia López-Franco, Francisco M. Goycoolea, Jaime Lizardi-Mendoza

**Affiliations:** 1 Centro de Investigación en Alimentación y Desarrollo A.C., Biopolímeros-CTAOA, Hermosillo 83304, Mexico; daniel.miramon85@gmail.com (D.A.M.-O.); waldo@ciad.mx (W.A.-M.); ecarvajal@ciad.mx (E.C.-M.); lopezf@ciad.mx (Y.L.L.-F.); 2 School of Food Science and Nutrition, University of Leeds, Leeds LS2 9JT, UK; f.m.goycoolea@leeds.ac.uk

**Keywords:** acemannan, gelation, aerogel, self-assembly, biological materials

## Abstract

The procedures to obtain two types of acemannan (AC) physical gels and their respective aerogels are reported. The gelation was induced by the diffusion of an alkali or a non-solvent, then supercritical CO_2_ drying technology was used to remove the solvent out and generate the AC aerogels. Fourier-transform infrared spectroscopic analysis indicated that alkali diffusion produced extensive AC deacetylation. Conversely, the non-solvent treatment did not affect the chemical structure of AC. Both types of gels showed syneresis and the drying process induced further volume reduction. Both aerogels were mesoporous nanostructured materials with pore sizes up to 6.4 nm and specific surface areas over 370 m^2^/g. The AC physical gels and aerogels enable numerous possibilities of applications, joining the unique features of these materials with the functional and bioactive properties of the AC.

## 1. Introduction

The acemannans (AC) are high molecular weight polysaccharides isolated from *Aloe vera* leaf pulp. This polysaccharide is known to be biodegradable, biocompatible and also could induce a beneficial immune response, making it appealing for numerous biomedical applications [1]. Several polysaccharides (e.g., chitosan, starch, alginate etc.) are known by their structural properties including gel formation capabilities. Some materials made of AC, such as membranes and sponge-like scaffolds [2,3], have been reported. However, there is limited information about the formation of gels with this type of polysaccharide. Gels are colloidal systems constituted of a liquid phase dispersed in spatially extended networks, usually assembled by synthetic or natural polymers joined by scattered connections [4]. Such junction points could be formed by either reversible physical associations or permanent chemical bonds [5,6]. When the liquid phase is removed without a generalized collapse of the gel-solid structure, a highly porous material (aerogel) could be produced. Aerogels are materials with the highest specific surface area available, mesoporous structures and relatively unique features [6,7,8]. Combining the functional properties of AC with the structural features of gels and aerogels could result in innovative materials with the potential use in several fields such as biomedicine, pharmaceutical, tissue engineering, food, etc. Herein, two novel procedures to obtain physical AC gels and the respective aerogels are reported, including their morphological and physical-chemical characteristics.

## 2. Materials and Methods

### 2.1. Materials

All solvents and chemicals used, such as acetone, ethanol (EtOH), liquid carbon dioxide (CO_2_), ammonium hydroxide (NH_4_OH) and sodium nitrate (NaNO_3_) were analytical grade reagents obtained from recognized suppliers. Type II water was used for all procedures unless stated otherwise.

### 2.2. AC Isolation

AC was isolated from *Aloe vera*, which was provided by a local plant nursery, following the method proposed by Campestrini et al. [9]. Briefly, the *Aloe vera* parenchyma (leaf pulp) was blended after removing the rind and sap. The obtained mixture was centrifuged at 9800× *g*, for 30 min, at 25 °C in a Heraeus Multifuge X3R Centrifuge (Thermo Fisher Scientific, Waltham, MA, USA). The polysaccharide fraction was recovered from the supernatant by precipitation, adding 4 volumes of EtOH (96%) and kept 24 h at 4 °C with gentle agitation. The precipitate, collected by centrifugation, was washed stepwise with EtOH at increasing concentrations (i.e., 70, 80, 90, and 100%), with a final rinse using acetone, then dried in vacuum oven at 60 °C for 24 h. The obtained dry matter was then dissolved in distilled water, dialyzed (regenerated cellulose membrane, 6000–8000 MWCO) against Type II water (water-exchange every hour for 6 h and then left overnight at 4 °C), frozen (−20 °C for 24 h), and then freeze dried for 72 h (collector temperature: −40 °C; vacuum: 0.08 mbar) in a Labconco Mobile Freeze Dryer Lyophilizer Vacuum 77,540 12 L (Labconco, Kansas City, MO, USA). 

### 2.3. AC Characterization

Contents of mannose, glucose, and galactose were determined by gas chromatography (GC) following the methodology proposed by Hemery et al. [10]. Neutral sugars were quantified as anhydro-sugars, after hydrolysis of the samples with sulphuric acid (1 M, 2 h and 100 °C) and transformation into alditol acetates. Anhydro sugar quantification was performed by gas chromatography (ELITE-225-30m capillary column; Perkin Elmer, Waltham, MA, USA) coupled to a flame ionization detector (FID) in an Agilent technologies 6890n gas chromatograph (Santa Clara, CA, USA), using hydrogen as the carrier gas and inositol (1 mg/mL) as the internal standard. The temperature of injection and detector were 220 and 260 °C, respectively. Monosaccharides were expressed as a percentage of neutral sugars quantified.

A multi-detector size exclusion chromatography system, including multi-angle laser light scattering, reflective index and viscosity Wyatt detectors (Dawn 8, Optilab T-rEX, ViscoStar II; Wyatt Technology Corporation, Santa Barbara, CA, USA, respectively) were used to determine the molecular weight and its distribution. The liquid chromatographic system Agilent 1260 Infinity II (Agilent technologies, Santa Clara, CA, USA) was fitted with an SB-G OHpak Shodex column (Shodex, New York, NY, USA). Up to 100 μL of filtered (0.45 μm nylon membrane; Supelco-Sigma Aldrich, St. Louis, MO)) AC solution (1 mg/mL) was injected in the column and eluted with sodium nitrate 0.1 M at 0.5 mL/min flow rate [11]. 

A 400 MHz Bruker Avance III spectrometer (Bruker, Billerica, MA, USA) was used to get the ^1^H nuclear magnetic resonance (NMR) spectrum of the extracted AC. For this, approximately 10 mg of AC were solubilized in 7.5 mL of D_2_O, then filtered through a 0.45 μm nylon membrane (Supelco-Sigma Aldrich). 

### 2.4. AC Gelation

Before gelation, the AC was dissolved in distilled water at 4 °C for 36 h to form a homogeneous solution at 1.5% (*w*/*v*). Measured quantities of AC solution (~100 mg) were poured into cylindrical molds. A number of filled molds were placed in closed chambers containing either ammonium hydroxide (M1) or acetone (M2). The vapor diffusion of the non-solvents induced the formation of AC physical gels for 48 h at room temperature [12].

The gels prepared by the M1 procedure were washed with water until neutral pH was reached. The water was gradually displaced by step rinses with acetone at increasing concentrations (i.e., 30, 40, 60, 70, 80, 90, and 100%) every 30 min with gentle stirring. The M2 gels were sequentially rinsed with 90, 95 and 100% acetone. The obtained gels were kept in pure acetone (acetogels) at room temperature until analysis or further processing.

### 2.5. AC Aerogel Formation

The supercritical drying technology was used to obtain AC aerogels from both gelation methods [8]. The displacement of the liquid phase of the acetogels was achieved using liquid CO_2_ in a pressurized reactor at 80 bar and a temperature of 40 °C. The drying procedure included three cycles of 15 min with 4.8 cc/min flow rate of liquid CO_2_ followed by 30 min without flow. Finally, the CO_2_ was slowly released (flow rate: ~5.0 cc/min) from the reactor. Dry nanostructured AC materials (aerogels) were obtained and stored under vacuum until analysis.

### 2.6. Gels and Aerogels Characterization

#### 2.6.1. Syneresis and Shrinkage

Gel syneresis and drying shrinkage of the AC aerogels was estimated following Equation (1).

(1) $$S=\frac{\left(Vi-Vf\right)}{Vi}*100$$

where *S* is the shrinkage, *Vi* and *Vf* are the volumes of gels before and after supercritical CO_2_ drying, respectively [8]. The longest diameter and length of imperfect geometry samples were used to calculate the volume. At least 25 monoliths were measured and comparisons were made using arithmetic means.

#### 2.6.2. Structural Analysis

Scanning electron microscopy (SEM) was used to evaluate the surface morphology of the AC aerogels. The images of samples were obtained in a Hitachi SU8000 (Tokio, Japan), using an accelerating voltage of 1.0 KeV.

Surface area and pore size of the aerogels were determined by gas sorption isotherms of N_2_, using a Nova 2200e analyzer (Quantachrome Instruments, Boynton Beach, FL, USA). Specific surface areas were determined from the BET equation and the average pore diameters were calculated by the BJH equation on the desorption branch of the isotherm. Before the assay, the aerogels were kept in vacuum overnight at 80 °C.

#### 2.6.3. Chemical Identity

Fourier-transform infrared spectroscopy (FTIR) was utilized to verify the chemical identity of the isolated AC, dry AC gels and aerogels. The samples were analyzed in a Nicolet iS50 FTIR spectrometer (Thermo Fisher Scientific, Waltham, MA, USA) using attenuated total reflectance (ATR) technique acquiring 32 scans with a resolution of 2 cm^−1^, at room temperature.

## 3. Results and Discussion

### 3.1. AC Characterization

The *Aloe vera* polysaccharide fraction was obtained using mild extraction procedures. The monosaccharide composition determined by GC-FID showed that mannose (88.78 ± 3.51%) was the predominant sugar present, followed by glucose (5.75 ± 0.17%) and galactose (4.94 ± 0.15%) with a molar proportion ratio of 18:1.2:1 (mannose-glucose-galactose). The presence of other sugars was below 1%. According to the carbohydrate composition, the extracted polysaccharide can be considered with confidence AC [11,13]. The physical-chemical characteristics of the isolated AC are included in Table 1.

FTIR spectrum of the isolated AC displays the characteristic bands to be expected from this polysaccharide: 1740 cm^−1^ (due to C=O stretching vibration), 1370 cm^−1^ (deformation of the H-C-OH bonds) and 1370 cm^−1^ (C–O–C asymmetric stretching vibration). Meanwhile, the ^1^H NMR spectrum shows the signals corresponding to the protons of the acetyl groups and the glycoside units (Figure S1, Supplementary Materials).

**Figure 1:**
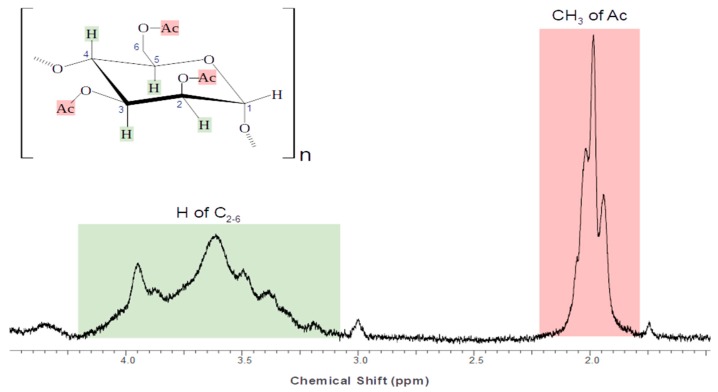
^1^H NMR spectrum (400 MHz) and peaks related to the monomeric glycoside unit (≈3.1 to 4.4 ppm; H_2_-H_6_) and Ac (acetyl) group (≈1.8 to 2.2 ppm; HAc) in AC.

The degree of acetylation of AC (DA) was determined using the ^1^H NMR data (Figure 1). The peak areas of the protons of the acetyl group (HAc; marked red in Figure 1) and of the protons linked to the C_2_-C_6_ of the glycoside unit (H_2-6_; marked green in Figure 1) were used to calculate the DA as follows [14]:

(2) $$DA=\frac{\left(HAc/3\right)}{\left(H_{2-6}/6\right)}$$

It has been reported that the carbons 2, 3 or 6 of mannose could be acetylated in AC [9]. Therefore, the maximum degree of substitution (i.e., acetylation) would be 3 (consistent with 2,3,6-tri-O-acetyl-(1-4)-α-d-mannopyranose). However, the reported DA for AC extracted from diverse aloe species are usually below the values of 1.4 [15,16]. The estimated DA value (1.3) in the AC sample falls in the higher end of this range. Similar DA values have been reported for acetylated glucomannans from *Aloe arborescens* [16].

The isolated AC molecular weight and polydispersity index, estimated by size exclusion chromatography with multi-angle laser light scattering (SEC MALLS), indicate that it is a high molecular-weight polysaccharide material with a heterogeneous size distribution, as expected. Diverse reports of molecular weight of AC fall in the range from 60 to 1300 kDa [9,17,18]. The variability observed in the reported DA and molecular weight parameters indicate that such AC characteristics depend on diverse factors related to the plant (i.e., age, place of growth, hydration, etc.) as well as the extraction procedures used [15].

### 3.2. AC Gelation and Aerogels Characterization

**Figure 2:**
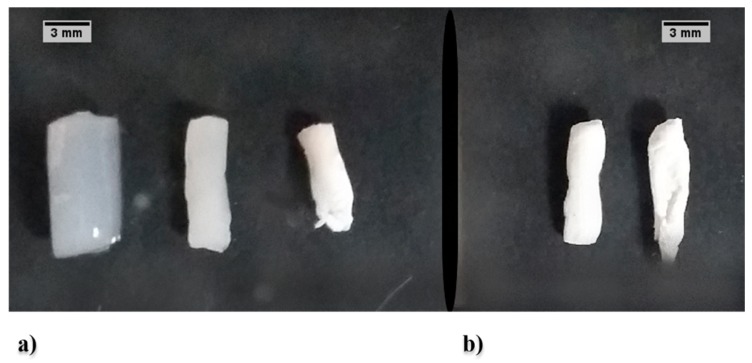
Acemannan gels. (**a**) AC hydrogel (left), acetogel (center) and aerogel (right) obtained by M1 process. (**b**) AC acetogel (left) and aerogel (right) obtained by M2 process.

AC gels were obtained using two different gelation promoting agents, namely M1 and M2 (Figure 2). The M1 type gels maintained most of the original cylindrical geometry (Figure 2a) preserving 78% of the original volume (i.e., 22% of syneresis). Just formed, the hydrogels looked translucent and swollen. Once transferred to acetone, they became opalescent and shrunk (~29%). The calculated overall volume reduction was ~51% of the original volume (i.e., casted AC solution). On the other hand, the M2 gels showed irregular cylindrical forms with white coloration (Figure 2b). In this case, the calculated volume reduction was ~61% of the original volume.

Syneresis is a phenomenon commonly observed in the diverse gel forming processes. The rate and extent of the volume reduction are determined by the molecular rearrangement involved in the particular gelation process. For the M1 procedure, the solvation of the AC molecules is gradually reduced by the action of the ammonium hydroxide inducing the hydrogel formation. The syneresis observed for the M1 AC hydrogels is apparently the result of a thermodynamic balance between coacervated portions of AC polymeric molecules, forming the crosslinking joints of the gel network, and the molecular fraction that remains hydrated. Conversely, in the M2 procedure, the non-solvent acetone dehydrates the AC molecules inducing self-assembly by entanglement that causes the formation of the three-dimensional network of the acetogels [12,19]. The molecular configuration at the end of the gelation process is affected by the capability of the fluid phase to form hydrogen bonds that cause partial folding of the polymer coils [20]. This also contributes to the formation of hydrogen bonding between separate chains of the polysaccharide decreasing the steric hindrance and reducing the volume occupied by the solid fraction of the gel [14]. A similar effect is observed throughout the solvent exchange in the M1 procedure, which may induce further interactions between the chains of the AC.

After both types of acetogels were obtained (M1 and M2), the supercritical CO_2_ drying procedure was carried out. The aerogels retained the original shape of the acetogel; however, an additional volume reduction took place during the CO_2_ supercritical drying. Mehling et al. reported similar findings of monolith-type aerogels prepared with different polysaccharides [7]. They observed different shrinkage rates ranging from 1 to 19% during supercritical drying. In covenant with these findings, in the present study, a ~10% (M2) and a ~17% (M1) of shrinkage caused by the drying process was obtained. According to these data, it can be presumed that the shrinkage depends on the type of polysaccharide, its physico-chemical properties, and the gelation method.

**Figure 3:**
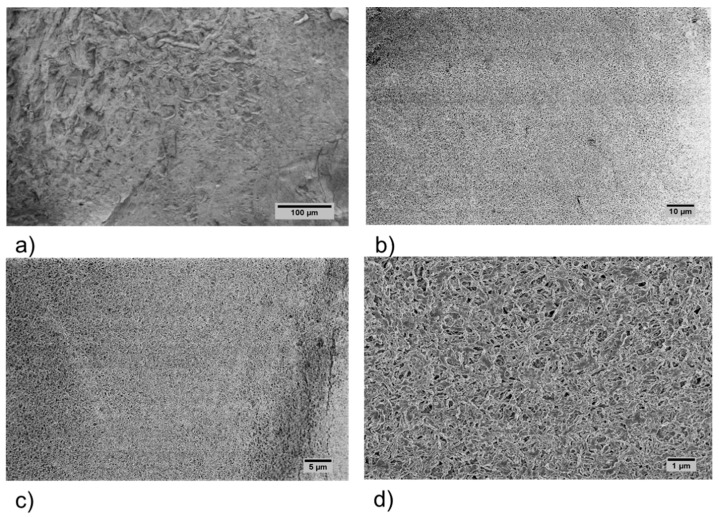
SEM micrographs of M1 acemannan aerogel at 200× (**a**), 1000× (**b**), 2000× (**c**) and 10,000× (**d**) magnification.

**Figure 4:**
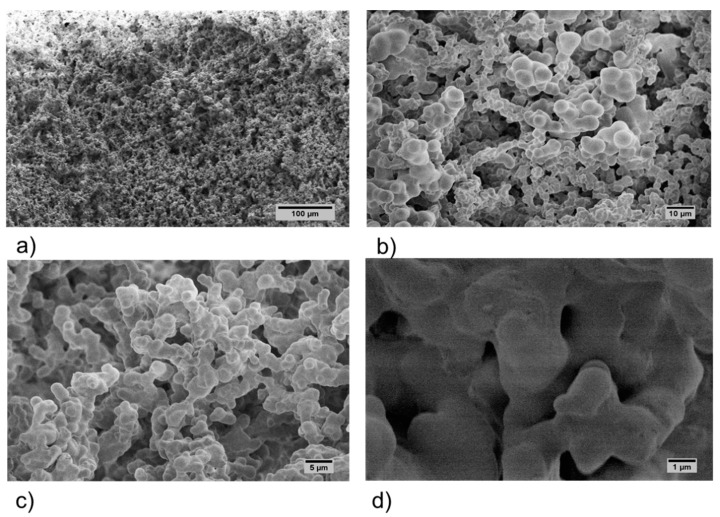
SEM micrographs of M2 acemannan aerogels at 200× (**a**), 1000× (**b**), 2000× (**c**) and 10,000× (**d**) magnification.

The surface morphology of the M1 and M2 aerogels (Figure 3 and Figure 4, respectively) was evaluated by SEM. The SEM analysis showed that aerogels displayed a nanostructured network formed with randomly arranged fibrils. A dense and apparently smooth surface with irregular depressions was observed at the lower magnification micrographs (Figure 3a–c), while an apparent mesoporous structure was distinguished at the highest magnification of the M1 aerogels (Figure 3d). On the other hand, an open porous structure was clearly present in M2 aerogels at the lowest magnification (Figure 4a). Such structure appeared to be constituted by several microfibrils joints at diverse points while forming numerous pores with size distribution below ~10 μm (Figure 4b–d).

The N_2_ adsorption-desorption isotherm studies allowed to assess the surface area and pore size of these materials. M1 aerogels exhibited a type IV isotherm with an H_2_-type hysteresis loop (Figure S2. Supplementary Materials), suggesting a well-built mesoporous structure. Their calculated specific surface area was ~370 m^2^/g with an average pore ratio estimated in ~6.4 nm. These data are comparable with other polysaccharide aerogels previously reported [6,7]. Conversely, the calculated specific surface area of the M2 aerogels was ~10 m^2^/g. Apparently, the solid structures formed by the M2 procedure exhibit a higher density morphology, resulting in lower surface area.

### 3.3. AC and Aerogels Chemical Identity

**Figure 5:**
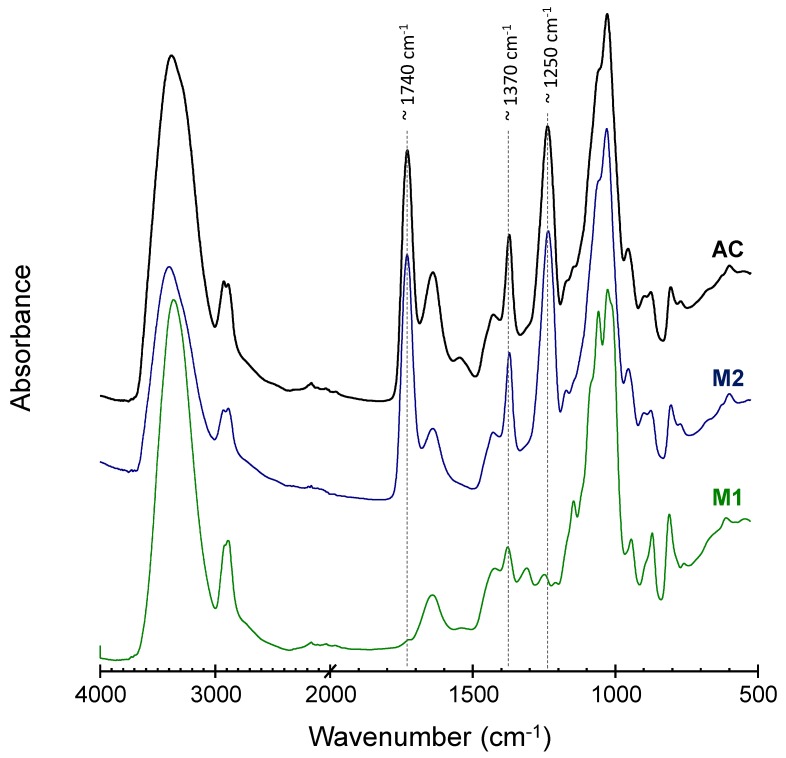
ATR-FTIR spectra of acemannan (AC), M1 and M2 aerogels.

FTIR spectroscopy was used to corroborate the chemical identity of the aerogels as compared with the pristine AC. The FTIR spectrum of AC (Figure 5) displays the characteristic bands observed in previous reports [14]. In the spectral region over 2500 cm^−1^, strong and broad band of OH stretching centred at 3380 cm^−1^, and the CH stretching band at 2900 cm^−1^ can be observed. In the middle region (1800–1200 cm^−1^), the strong bands associated with C=O and C-O-C stretching (1740 and 1250 cm^−1^, respectively) could be appreciated. These bands are related to the presence of the acetyl groups. There are also two medium bands, centered at 1640 and 1430 cm^−1^, associated to the vibration of the O–C–O bonds, along with a band at 1370 cm^−1^ due to the deformation of the H–C–OH bonds. These bands can be related to the glycoside units of the acemannan. The region below 1200 cm^−1^ contains a complex combination of bands known as the “fingerprint” zone that has been associated with the carbohydrate ring and skeletal vibrations. The FTIR spectrum of M2 aerogel closely resembles that of the original AC spectrum, thus, indicating that gelling and drying processes do not cause chemical changes. On the other hand, there are remarkable differences between the spectra of AC and M1 aerogel due to the reduction to a minimum of a couple of bands associated to the acetyl group (centered at 1740 and 1250 cm^−1^).

**Figure 6:**
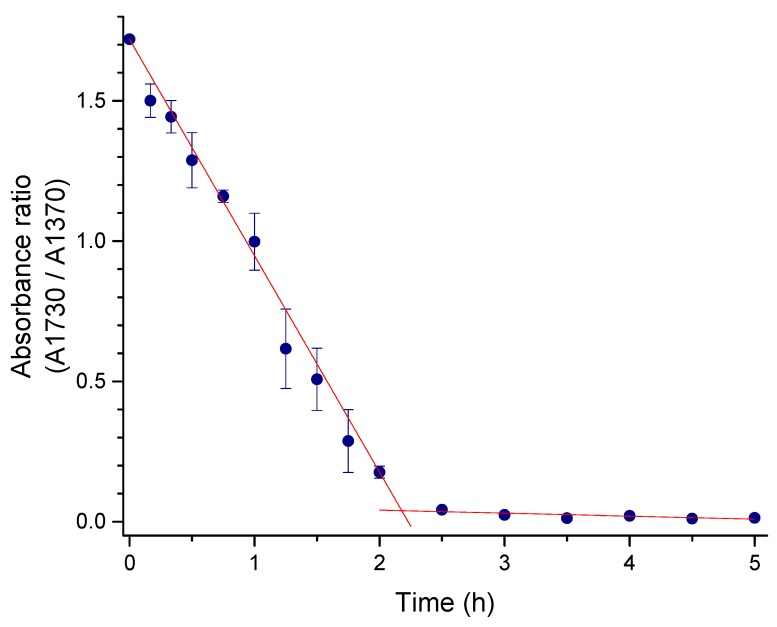
Selected bands absorbance ratio (A1740/A1370) of AC through the M1 treatment time.

The possible deacetylation produced by the diffusion of NH_4_OH into the AC solution was evaluated by measuring the acetyl related infrared bands of AC treated with NH_4_OH vapor at varying times. The absorbance vs. time series data was analyzed by a trend analysis. According to the fitted trend equation, the signals with greater and minor change were selected as the probe and reference bands, at 1740 and 1370 cm^−1^, respectively. The absorbance of the band at 1740 cm^−1^ was measured using a baseline from the inflection at 1790 to 1690 cm^−1^; similarly, the absorbance of the 1370 cm^−1^ band was measured from the baseline drawn from the inflections at 1500 to the 1340 cm^−1^. The behavior of the absorbance ratio of the selected bands (A1740/A1370) is shown in Figure 6. Since the 1740 cm^−1^ band corresponds with the C=O stretching vibration, it is associated with the degree of acetylation of the AC. The observed reduction within the first two hours of the M1 treatment is related to a deacetylation process. There are previous reports indicating that AC could be deacetylated by alkaline treatments [9]. Similarly, the band related to the C–O–C asymmetric stretching vibration for aliphatic esters (1250 cm^−1^) also decreased, whereas, the bands associated with the hydroxyl group apparently increased. These data indicate that the gelation in M1 was probably promoted by alkaline deacetylation of the AC. In the proposed reaction, hydroxyl groups worked as nucleophiles striking the carbonyl carbon (electron-deficient) of the acetyl group and acetate was instantaneously liberated, resulting in the substitution of the acetyl group with a hydroxyl group [14]. The gel formation could be related to the removal of the acetyl moieties along the AC chain, reducing steric hindrance effects with a concomitant increase of intermolecular interactions, mainly, hydrogen bonding.

## 4. Conclusions

Being AC a polymer with high potentialities in biomedicine, these results demonstrated that it is possible to prepare two types of AC gels and aerogels with different structural and chemical features. One type of gel was produced through molecular rearrangement and self-assembly after extensive AC deacetylation. AC gelation was also achieved by molecular self-assembly induced with a non-solvent (acetone) vapor diffusion. With supercritical drying technology, AC aerogels with high surface and porosity can be obtained after gelation with both methods described. The gels obtained by these procedures have the potential to be employed in several fields since they are high-porous materials with high and huge specific surface areas, making them suitable for diverse adsorption/absorption of substances such as drugs, cell components, contaminants, liquids, etc. Ongoing studies will provide further information on the gelation mechanisms, functional and biological properties of these biopolymeric materials.

## Figures and Tables

**Table 1:** Physical-chemical characteristics of AC.

Characteristic	Value	Unit
DA ^a^	1.31 ± 0.01	
dn/dc ^b^	0.16 ± 0.00	
Mw ^c^	1500	kDa
Mn ^c^	870	kDa
Ip ^c^	1.9	

^a^ Degree of acetylation (DA) determined by ^1^H NMR spectroscopy. ^b^ Differential refractive index (dn/dc) of AC. ^c^ Molecular weight distribution parameters determined by SEC-MALLS: Weight-average molecular weight (*M*_w_); Number-average molecular weight (*M*_n_) and polydispersity index (Ip = *M*_w_/*M*_n_) [11].

## Supplementary Materials

The following are available online at http://www.mdpi.com/2073-4360/11/2/330/s1, Figure S1: 1H NMR spectrum of AC., Figure S2: Adsorption isotherms of acemannan aerogels. (**A**) M1, (**B**) M2.

## References

[1] Kumar S., Tiku A.B. (2016). Immunomodulatory potential of acemannan (polysaccharide from Aloe vera) against radiation induced mortality in Swiss albino mice. Food Agric. Immunol..

[2] Silva S.S., Caridade S.G., Mano J.F., Reis R.L. (2013). Effect of crosslinking in chitosan/aloe vera-based membranes for biomedical applications. Carbohydr. Polym..

[3] Silva S.S., Oliveira M.B., Mano J.F., Reis R.L. (2014). Bio-inspired Aloe vera sponges for biomedical applications. Carbohydr. Polym..

[4] Berger J., Reist M., Mayer J.M., Felt O., Peppas N.A., Gurny R. (2004). Structure and interactions in covalently and ionically crosslinked chitosan hydrogels for biomedical applications. Eur. J. Pharm. Biopharm..

[5] Zhang C. (2008). Interplay between long-range and short-range interactions in polymer self-assembly and cell adhesion. Ph.D. Thesis.

[6] García-González C.A., Alnaief M., Smirnova I. (2011). Polysaccharide-based aerogels - Promising biodegradable carriers for drug delivery systems. Carbohydr. Polym..

[7] Mehling T., Smirnova I., Guenther U., Neubert R.H.H. (2009). Polysaccharide-based aerogels as drug carriers. J. Non-Cryst. Solids.

[8] Wang X., Zhang Y., Jiang H., Song Y., Zhou Z., Zhao H. (2016). Fabrication and characterization of nano-cellulose aerogels via supercritical CO2 drying technology. Mater. Lett..

[9] Campestrini L.H., Silveira J.L.M., Duarte M.E.R., Koop H.S., Noseda M.D. (2013). NMR and rheological study of Aloe barbadensis partially acetylated glucomannan. Carbohydr. Polym..

[10] Hemery Y., Holopainen U., Lampi A.M., Lehtinen P., Nurmi T., Piironen V., Edelmann M., Rouau X. (2011). Potential of dry fractionation of wheat bran for the development of food ingredients, part II: Electrostatic separation of particles. J. Cereal Sci..

[11] Escobedo-Lozano A.Y., Domard A., Velázquez C.A., Goycoolea F.M., Argüelles-Monal W.M. (2015). Physical properties and antibacterial activity of chitosan/acemannan mixed systems. Carbohydr. Polym..

[12] Montembault A., Viton C., Domard A. (2005). Rheometric Study of the Gelation of Chitosan in Aqueous Solution without Cross-Linking Agent. Biomacromolecules.

[13] Ni Y., Turner D., Yates K.M., Tizard I. (2004). Isolation and characterization of structural components of Aloe vera L. leaf pulp. Int. Immunopharmacol..

[14] Chokboribal J., Tachaboonyakiat W., Sangvanich P., Ruangpornvisuti V., Jettanacheawchankit S., Thunyakitpisal P. (2015). Deacetylation affects the physical properties and bioactivity of acemannan, an extracted polysaccharide from Aloe vera. Carbohydr. Polym..

[15] Ni Y., Yates K.M., Tizard I., Reynolds T. (2004). Aloe polysaccharides. Aloes: the genus Aloe.

[16] Wozniewski T., Wolfgang B., Franz G. (1990). Isolation and structure analysis of a glucomannan from the leaves of Arborescens var. Miller. Carbohydr. Res..

[17] Femenia A., Sánchez E.S., Simal S., Rosselló C. (1999). Compositional features of polysaccharides from Aloe vera (Aloe barbadensis Miller) plant tissues. Carbohydr. Polym..

[18] Turner C.E., Williamson D.A., Stroud P.A., Talley D.J. (2004). Evaluation and comparison of commercially available Aloe vera L. products using size exclusion chromatography with refractive index and multi-angle laser light scattering detection. Int. Immunopharmacol..

[19] Montembault A., Viton C., Domard A. (2005). Rheometric study of the gelation of chitosan in a hydroalcoholic medium. Biomaterials.

[20] Subrahmanyam R., Gurikov P., Dieringer P., Sun M., Smirnova I. (2015). On the Road to Biopolymer Aerogels—Dealing with the Solvent. Gels.

