# Supplementary material for: Acemannan Gels and Aerogels
Source: Polymers (Basel). 2019 Feb 14;11(2):330. doi: 10.3390/polym11020330 (PMC6419202; doi:10.3390/polym11020330)
Supplement: Supplementary file 1 [file polymers-11-00330-s001.pdf]

polymers

Communication

Acemannan gels and aerogels

Daniel Alonso Miramon-Ortiz[1], Waldo Argüelles-Monal[1], Elizabeth Carvajal-Millan[1], Yolanda Leticia López-Franco[1], Francisco M. Goycoolea[2], and Jaime Lizardi-Mendoza[1,*]

[1] Centro de Investigación en Alimentación y Desarrollo A.C., Biopolímeros - CTAOA; (D.A. M-O., daniel.miramon85@gmail.com; W. A-M, waldo@ciad.mx; E. C-M, ecarvajal@ciad.mx; Y.L. L-F, lopezf@ciad.mx; J. L-M, jalim@ciad.mx)

[2] University of Leeds, School of Food Science and Nutrition; (F.M. G., f.m.goycoolea@leeds.ac.uk)

* Correspondence: jalim@ciad.mx

Received: date; Accepted: date; Published: date

Supplementary Material

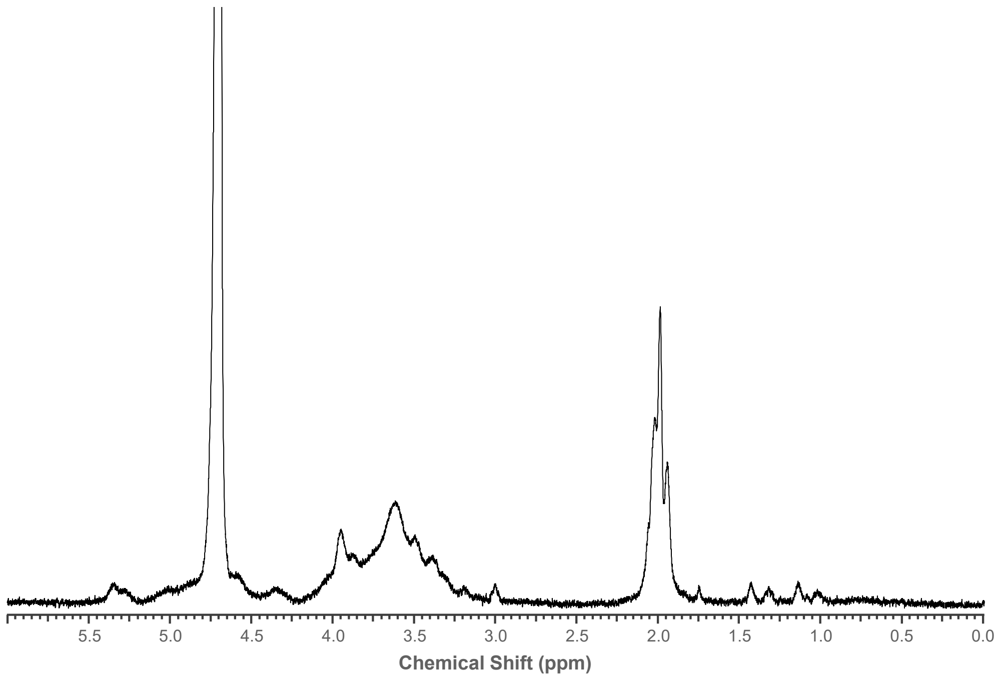

Figure S1. ^{1}H NMR spectrum of AC.

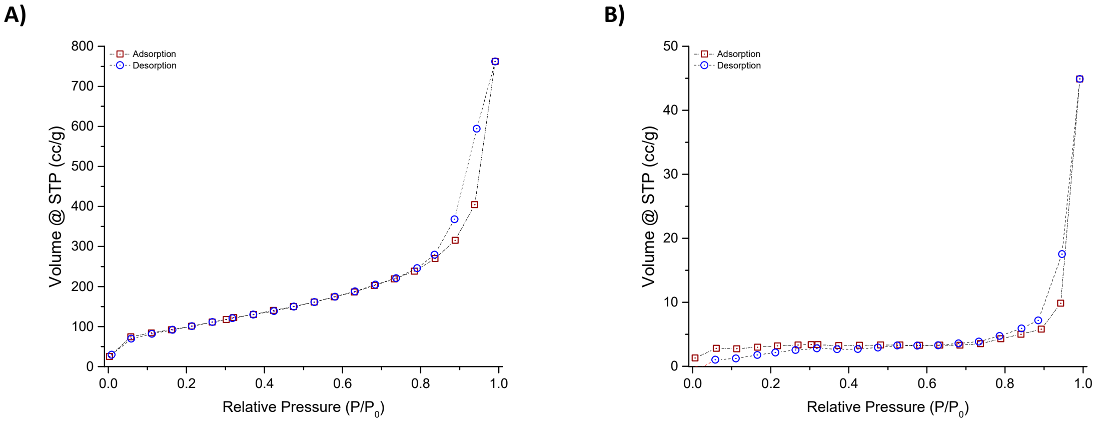

Figure S2. Adsorption isotherms of acemannan aerogels. **A**) M1, **B**) M2.

© 2019 by the authors. Submitted for possible open access publication under the terms and conditions of the Creative Commons Attribution (CC BY) license (http://creativecommons.org/licenses/by/4.0/).
